# Targeting Non-Oncogene Addiction for Cancer Therapy

**DOI:** 10.3390/biom11020129

**Published:** 2021-01-20

**Authors:** Hae Ryung Chang, Eunyoung Jung, Soobin Cho, Young-Jun Jeon, Yonghwan Kim

**Affiliations:** 1 Department of Biological Sciences and Research Institute of Women’s Health, Sookmyung Women’s University, Seoul 04310, Korea; jey0605@sookmyung.ac.kr (E.J.); sbsjjh0119@naver.com (S.C.); 2 Department of Integrative Biotechnology, Sungkyunkwan University, Suwon 16419, Korea; jeon2020@skku.edu

**Keywords:** non-oncogene addiction, DNA damage response, DNA repair, cancer therapy

## Abstract

While Next-Generation Sequencing (NGS) and technological advances have been useful in identifying genetic profiles of tumorigenesis, novel target proteins and various clinical biomarkers, cancer continues to be a major global health threat. DNA replication, DNA damage response (DDR) and repair, and cell cycle regulation continue to be essential systems in targeted cancer therapies. Although many genes involved in DDR are known to be tumor suppressor genes, cancer cells are often dependent and addicted to these genes, making them excellent therapeutic targets. In this review, genes implicated in DNA replication, DDR, DNA repair, cell cycle regulation are discussed with reference to peptide or small molecule inhibitors which may prove therapeutic in cancer patients. Additionally, the potential of utilizing novel synthetic lethal genes in these pathways is examined, providing possible new targets for future therapeutics. Specifically, we evaluate the potential of TONSL as a novel gene for targeted therapy. Although it is a scaffold protein with no known enzymatic activity, the strategy used for developing PCNA inhibitors can also be utilized to target TONSL. This review summarizes current knowledge on non-oncogene addiction, and the utilization of synthetic lethality for developing novel inhibitors targeting non-oncogenic addiction for cancer therapy.

## 1. Introduction

The average lifespan of humans is increasing as stated by the World Health Organization (WHO), and cancer incidence rates rise in tandem [1]. As technology has advanced cancer therapies have diversified. Now with NGS and systems analysis, “personal medicine” has evolved into “precision medicine” as initiated by the former United States president Barack Obama in 2015 [2,3]. According to the United States National Library of Medicine, precision medicine is defined as "an emerging approach for disease treatment and prevention that takes into account individual variability in genes, environment, and lifestyle for each person." [2,3]. With precision medicine, medical experts can cater treatments to the individual’s disease profile with high accuracy, resulting in better patient outcomes. The “Hallmarks of cancer” have served an essential role in understanding tumorigenic mechanisms and provided a roadmap for developing different treatment regimens [4,5]. Surgery, chemotherapy, radiation therapy, as well as an increasing number of targeted therapies and immunotherapies have provided a plethora of options for patients with different types and stages of cancer. In the New England Journal of Medicine’s review on 200 years of cancer research, DeVita et al. elegantly displayed the milestones of cancer research, especially the timeline of pivotal events in cancer treatment development [6]. Medical research has not stopped since, and by identifying and developing biomarkers and diagnostic kits to accurately identify cancers in an earlier stage, a variety of therapeutic options can be offered early resulting in improved outcomes [7,8,9,10,11,12].

In developing these therapeutic and diagnostic options, translational research and basic biology has played a critical role in identifying key pathways [6,8,13]. In establishing the hallmarks of cancer, driver and passenger mutations were identified, and cancer cell biology was further exposed, including tumor microenvironment and different forms of addiction such as the Warburg effect and oncogene addiction [14,15,16,17,18,19]. These hallmarks elucidated the differential gene expression and alteration of normal cellular functions into tumorigenic mechanisms and continues to identify potential therapeutic targets. Of the unique characteristics found in cancer cells, non-oncogene addiction is another mechanism that is commonly targeted for developing cancer treatments.

The term “oncogene addiction” was first coined by Bernard Weinstein, describing the physiological dependency of cancer cells to continuous activation or overexpression of oncogenes [14,20,21]. The Cancer Quest website of the Winship Cancer Institute of Emory University provides a comprehensive list of oncogenes and their related cancer (https://www.cancerquest.org/cancer-biology/cancer-genes#table). Many drugs targeting these oncogenes are being developed and have been FDA approved, including trastuzumab for *HER2*, sorafenib for *BRAF*, gefitinib for *EGFR*, imatinib for *ABL*, as well as *KIT*, *PDGFR*, and bevacizumab targeting *VEGF* [20,21,22].

As mentioned above, the overexpression and/or alterations of these oncogenes often becomes a major driver of cancer cell proliferation. On the other hand, there are genes that are not oncogenic, but are essential for tumor cell survival. Often times, normal cells may not be as dependent on these genes or pathways, and yet they are essentially required for cancer cell survival [23,24,25,26]. Thus, the term “non-oncogene addiction” was coined, due to the fact that many of these genes that are often tumor suppressor genes and are critical for cancer cell survival. Although one of the functions of tumor suppressors is involved in is preventing cell cycle progression after DNA damage, several genes involved in these pathways are often found overexpressed in cancer cells whose survival is dependent on cell cycle progression [27,28]. Bartkova et al. revealed, often times, DDR precedes p53 mutation, which is one of the major factors affecting tumor development [27]. Therefore, genes involved in DDR, replication stress and cell cycle, provide potential therapeutic targets while many also show synthetic lethality with known oncogenes. Luo et al. suggest two approaches to utilizing non-oncogenic addiction for treatment development [24]. Because cancer cells are under many cellular stresses compared to normal cells, these stress support systems can be sensitized or overloaded, leading to cell death. DDR pathways and replication stress are some examples. Mutations in genes in these pathways can lead to the accumulation of DNA damage, which can cause enormous stress, sensitizing cancer cells to therapeutics.

**Figure 1:**
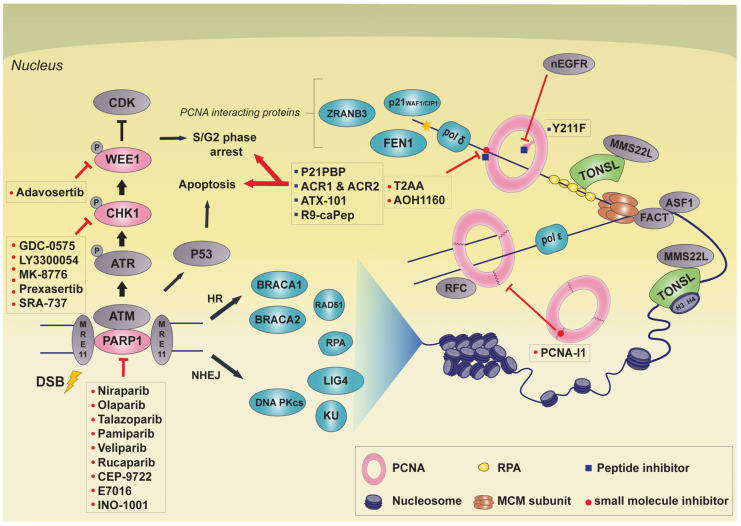
Summary of the major non-oncogene addiction related cancer therapy target proteins and its inhibitors discussed in this review. PARP1, CHK1, and Wee1 are involved in DNA DSB recognition and repair as well as cell cycle regulation. Similarly, TONSL is involved in DNA DSB repair, stalled replication fork repair and proper chromatin formation via its interaction with histone. PCNA is involved in a wide range of cellular processes; DNA replication, DNA damage repair, and cell proliferation. PCNA inhibitors prevent PCNA from binding with interacting protein, being phosphorylated or bound to chromatin. The workings of these inhibitors impede genome integrity so that can halt cell growth or lead to apoptosis. The inhibitors for each protein are shown, and their types are distinguished by markers. The red circles indicate small molecule inhibitors and the blue squares indicate peptide inhibitors.

There are many small molecule DDR inhibitors targeting proteins like PARP, CHK1, ATR, and Wee1. In this review, we present TONSL as a potential novel target for cancer therapy. We discuss PCNA inhibitors that may share similar aspects as TONSL inhibitor development, as both proteins do not harbor enzymatic function, but are known scaffolding proteins. Furthermore, four well-established cancer therapeutic targets that are involved in DDR and replication stress will be discussed (Figure 1). In addition, the newest small molecule drugs targeting these genes that are currently undergoing clinical trials will be discussed to provide a current update on targeted therapies involving non-oncogene addiction.

## 2. Strategies Targeting PCNA

Proliferating cell nuclear antigen (PCNA) is a scaffold protein associated with various pathways, that is vital for diverse functions in DNA replication, DNA repair, gene expression, and epigenetic regulation [29,30,31,32]. As PCNA is an important protein for cancer cell growth and survival, PCNA represents a potential target for anticancer therapy. In comparison to nonmalignant cells, accumulated evidence has shown that PCNA is overexpressed or posttranslationally modified in malignant cells [33,34,35,36,37], making it widely used as a biomarker in the diagnosis and prognosis of various cancers [38,39,40,41,42]. This is because certain cancer cells are addicted to essential pathways, where PCNA is active, and thus inhibition of PCNA can be used as a potential anticancer therapy. In addition, cancer cells have genome instability due to the accumulation of gene mutations. This means PCNA must be more active in cancer cells than nonmalignant cells in order to maintain genome integrity. PCNA is also involved in a variety of other processes, as DNA replication overlaps with a wide range of cellular processes related to DNA repair and cell growth [43,44]. Given these data, it was proposed that chemotherapy with genotoxic agents inducing DNA damage could be more effective when combined with PCNA inhibition [45]. PCNA has a unique structure; three PCNA monomers form a homotrimer by head-to-tail joining, which is eventually formed into a ring structure [46,47]. The inside of the ring structure is positively charged by the alpha helix and is in a position to look perpendicular to the phosphate backbone of DNA. The outer surface of PCNA consists of beta sheets and long interdomain-connecting loops (IDCL), responsible for PCNA interaction with various proteins [48]. This unique ring structure allows PCNA to encircle the double helix structure of DNA and slide freely on it [49]. The ring of PCNA then acts as a platform that tethers polymerases tightly and interacts with co-factors related to DNA replication and synthesis [49]. Inhibitors targeting PCNA using these structural properties are mainly divided into two groups; those that block docking sites to prevent PCNA from binding other proteins, and those that interfere with structure formation of PCNA homotrimers.

### 2.1. Peptide Inhibitor: Peptide Mimetics

PCNA is associated with various proteins through IDCL and most of those binding partners interact through specific motifs, such as PCNA-interacting protein (PIP) boxes or AlkB homolog 2 PCNA-interacting motif (APIM) [50,51,52]. p21^WAF1/CIP1^ is tightly associated with PCNA through its PIP box located on the C-terminus [50,53]. In vitro assays showed that the p21 and PCNA interaction impairs replicative DNA synthesis [54,55,56], however, the functional relevance of this interaction in vivo is still controversial [57]. Using peptide mapping analysis, Warbrick et al. identified the region of p21 critical for PCNA interaction, and named it p21WAF1 PCNA-binding peptide (p21PBP) [53]. The p21PBP, KRRQTSMTDFYHSKRRLIFS (amino acids 141–160 of p21), contains PIP-box residues, QTSMTDFY, and is capable of interacting with PCNA [53,58]. p21PBP competes with other PCNA binding partners, including DNA polymerases and, thus, inhibits PCNA function during DNA replication and repair, resulting in cell cycle arrest during S-phase [53,58,59]. The protein-protein inhibition property of p21PBP has been enhanced by peptidomimetic-based approaches. Functional potency of a peptide ligand can be enhanced by replacement of some amino-acid residues or harnessing alternative covalent modification. Wegener et al. took advantage of a peptidomimetics strategy to enhance the inhibition properties of p21PBP, including selectivity, potency, and biostability. They developed peptide mimetics of p21PBP, ACR1, and ACR2, finding that the ACR2 showed enhanced biostability and higher selectivity [60]. Despite their excellent binding affinity and stability, these peptides have not yet been reported on in clinical trials to date.

Another possible strategy could be to target the APIM motif to inhibit PCNA binding and thus impair cellular pathways mediated by these protein interactions. A number of DNA damage repair proteins interact with PCNA through APIM [51,52]. For example, it was reported that DNA repair proteins, such as ZRANB3 and FBH1, which are important to ICL repair, interact with PCNA through APIM [61,62]. During treatment with genotoxic reagents for cancer therapy, DNA damage accumulates in cancer cells which eventually leadings to cell death. However, if the interaction between PCNA and ZRANB3 or FBH1 is hindered, DNA repair will be interrupted, which results in more DNA damage. Given these data, it is likely that peptides targeting the APIM motif will be more effective when combined with genotoxic drugs which induce DNA lesions. ATX101 is a peptide type PCNA inhibitor targeting the APIM-interacting region [63]. The anticancer efficacy of this inhibitor was promising when combined with other drugs in multi-myeloma, bladder cancer cell lines, and prostate cancer cells [63,64,65]. Treatment with ATX101 blocks the interaction between DNA repair proteins and PCNA, impeding genome integrity in genotoxic stress situations; the upregulation of PCNA in malignant cells makes them more vulnerable than nonmalignant cells to PCNA inhibition [63]. Although APIM peptide injection is reported to have some effect on cell growth depending on cell type and dose, most APIMs are known to have major functions in the DNA damage response and repair protein [63,65,66,67]. A combination of chemotherapy with genotoxic agents, such as cisplatin, has proven more effective than chemotherapy alone [67,68]. Nonmalignant cells do not react as sensitively as malignant cells under stressful conditions. During cellular stress, the malignant cell is excessively modified by posttranslational modification (PTM), resulting in an APIM interaction mediated protein responses [63]. After combination treatment, tumor sizes were significantly decreased, possibly by seemed to affecting cell development and apoptosis signaling pathways [65]. When combined with docetaxel, APIM targeted peptides resulted in decreased tumor volumes compared to docetaxel alone. Additionally, combination treatment was effective in suppressing tumor regrowth. Encouragingly, there were no side effects from docetaxel, such as weight loss, in the combination treatment group [65]. This shows PCNA inhibitors combined with other treatment may not only improve anti-cancer therapy effects but also compensate for the side effects of other anti-cancer drugs.

One advantage of PCNA as a therapeutic target is based on the cancer-associated PCNA isoform (caPCNA), which appears to be prominently expressed in cancer cells and tumor tissues [69,70]. It was reported that the L126-Y133 region of caPCNA is differently modified by posttranslational modification in cancer cells, which allows the region more access to binding partners [69,71]. The protein binding accessibility for the L126-Y133 in cancer cells is different from normal cells and thus targeting the modified L126-Y133 using peptides showed a distinct toxicity [72]. The L126-Y133 region exists within the interconnector domain, where PCNA interacts with other proteins [71]. Gu et al. developed the R9-caPep peptide consisting of nine arginine residues and L126-Y133, which effectively inhibited PCNA and FEN1 or LIG1 interactions. As FEN1 and LIG1 are implicated in Okazaki fragment processing during the S-phase, treatment with R9-caPep in cell culture leads to stalled replication forks and cell cycle arrest [72]. In addition, it was found that treatment with R9-caPep leads to DNA damage accumulation as PCNA also plays a role in DNA repair pathways. Specifically, R9-caPep impairs homologous recombination (HR), but not in non-homologous end joining (NHEJ) pathways. As HR is the major DNA double strand break pathway in S-phase, it was proposed that R9-caPep mediated cell cycle arrest might cause HR defects. Interestingly, R9-caPep is most effective on *MYCN* overexpressed neuroblastoma cells [72]. Later it turned out that *MYCN*-amplified NB cells display higher replicative stress markers and conferring additional replicative stress by R9-caPep synergistically sensitizes the NB cells [73]. These findings suggest that further studies will be required to identify cancer types that are sensitive to R9-caPep, which will promote the application of R9-caPep as viable part of cancer therapy.

In triple-negative breast cancer patients, Tyrosine 211 (Y211) PCNA phosphorylation is known to be related to cancer proliferation and a lowered survival rate [35]. In previous clinical research, EGFR TKI (Epidermal growth factor receptor tyrosine kinase inhibitor) did not have sufficient therapeutic effects on drug-resistant cancers, even though EGFR expression was upregulated [74,75]. To overcome the cancer’s resistance to therapy, further studies have been carried out investigating the inhibition of PCNA, downstream of EGFR activation. Phosphorylation of the PCNA Y211 site is known to be highly stable in chromatin-bound conditions and to increase activity during DNA replication and DNA repair [35]. Nuclear EGFR (nEGFR), which functions in the nucleus and acts as tyrosine kinase to phosphorylate PCNA, specifically phosphorylates chromatin-bound PCNA. Phosphorylated PCNA maintains its stability during genome replication and DNA repair while also playing a central role in cell growth [35,76]. In an attempt to inhibit PCNA Y211 phosphorylation by nEGFR, the Y211F peptide was fused to the nuclear penetrating peptide TAT. Y211F peptide covers 12 amino acids flanking the Y211, replacing the tyrosine (Y) with phenylalanine (F) [77]. Treatment with the Y211F peptide led to inhibition of DNA synthesis, cell cycle arrest, and cancer cell death [78]. In vivo studies demonstrated that the Y211F peptide resulted in decreased tumor growth in a xenograft mouse model, suggesting that Y211F peptide could be an applicable alternative strategy for cancer therapy [76]. TAT-based Y211F cell-penetrating PCNA peptide (CPPP) has been reported to be effective in suppressing the proliferation of TNBC (triple negative breast cancer) cells that have become treatment resistant, especially to EGFR TKI [77]. At the molecular level, Y211F CPPP treatment impaired the interaction between nEGFR and PCNA in a competition-based manner. Indeed, treatment with Y211F peptide results in reduction of PCNA Y211 phosphorylation, defects in cell proliferation, and cell death. Not only that, when combined with TKIs, the Y211F peptide synergistically sensitized the TKI resistant cancer cells [77], demonstrating that Y211F CPPP might be a potential cancer therapy option for TKI resistant cancers.

### 2.2. Small Molecule Inhibitors

Small molecules could also be used to effectively inhibit PCNA function. Punchihewa et al. developed high affinity small molecules targeting PCNA [79]. They performed a chemical library screening and discovered that a thyroid hormone, 3,3’,5-triiodothyronine (T3) can prevent the interaction between PCNA and PIP box peptides. Crystal structure of the T3 showed that this molecule directly binds to the same motif of PCNA where the PIP box sequence peptide interacts, suggesting that T3 might be able to abolish PCNA binding [79]. However, having strong thyroid hormone activity, the T3 itself is not suited for clinical use in cancer cases. Therefore, to develop a chemical that does not have hormone activity, Punchihewa et al. designed a number of T3 derivatives before finally developing a fine-tuned small molecule PCNA inhibitor, T2 amino alcohol (T2AA) [79]. T2AA does not have thyroid hormone activity, shows higher affinity to PCNA than T3, and typically inhibits the binding of the high affinity p21 protein. T2AA also prevents chromatin bound PCNA from interacting with DNA polymerase δ, making it another potential therapeutic candidate [79]. In further studies, Punchihewa and colleagues found that treatment with T2AA leads to enhanced activation of the DNA damage response, cell cycle arrest in S-phase, and apoptosis. They demonstrated that in the presence of T2AA, PCNA failed to recruit translesion DNA polymerases, mediated by PIP box peptides, to the sites of DNA damage. This prevented repair to the lesions, resulting in the accumulation of more DNA damage [79]. These findings raised the possibility that combined use of T2AA with genotoxic reagents could have synergistic chemotherapeutic effects. As expected, combined treatment of T2AA and cisplatin showed enhanced cancer cell death [79], and thus combination treatment strategies have been shown as potentially useful experimentally.

PCNA is a ring-structured homotrimer and monomers are associated by head to tail interaction. In order for the homotrimer PCNA to perform various functions in the cell, the PCNA must be loaded onto chromatin by replication factor C complex (RFC) [46,47,80], which allows PCNA to participate in the DNA replication and repair pathways [30]. In the process of loading onto chromatin, the association of PCNA monomers is disengaged, leading to an opening of the ring, the PCNA homotrimer sits on the chromatin, followed by re-formation of the PCNA ring structured homotrimer [81]. In silico analysis and structural similarity search of the ZINC chemical database analysis identified PCNA-I1 as potentially interacting with PCNA in an inhibitory way [82]. It was suggested that PCNA-I1 binds to the junction of the head-to-tail interaction sites between PCNA monomers, and thus stabilizes the PCNA homotrimer resulting in insensitivity to RFC interactions [83,84]. Biochemical analysis showed that treatment with PCNA-I1 promotes SDS-refractory PCNA trimer formation while few PCNA trimers were observed in SDS-PAGE with mock treatment. In addition, the loading of PCNA onto chromatin was significantly reduced in the presence of PCNA-I1 in a dose and time dependent manner [82], demonstrating that PCNA-I1 is capable of inhibiting the functions of PCNA. At the cellular level, treatment with PCNA-I1 showed similar effects as siRNA-mediated PCNA depletion. PCNA-I1 treatment leads to inhibition of replication, cell cycle arrest during S-phase, and suppression of cancer cell growth [82]. As the half maximal inhibitory concentration (IC_50_) of PCNA-I1 in cancer cell growth (IC_50_, 0.17 ± 0.07 µM) is low enough compared to normal cell growth (IC_50_, 1.60 ± 0.36 µM), PCNA-I1 together with other PCNA-Is can be used as potential PCNA-targeted cancer therapies [82,84].

A good small molecule for cancer therapy is a compound that is specific to cancer cells and widely applicable to various types of tumors. Unlike PCNA in normal cells, cancer cells have distinctive features that distinguish them from normal cells. Among the features, posttranslationally modified L126-Y133 of caPCNA could be one potential target for small molecule development. The caPCNA L126-Y133 region has structural features that are more accessible to other interacting proteins [69,70]. Gu et al. performed virtual screening to select small molecules targeting the L126-Y133 of caPCNA and identified AOH39, and its derivative AOH1160, were likely to interfere with the interaction between caPCNA and T3 [85]. As expected, treatment with both chemicals in vitro resulted in impairment of DNA replication, DNA repair, induced the accumulation of DNA damage, and caused cell cycle arrest during S-phase, which lead to cell growth inhibition. AOH1160 is toxic to various cancer cells with an IC_50_ ranging from 0.11 µM to 0.53 µM, but is relatively nontoxic to nonmalignant cells with an IC_50_ around 5 µM. Therefore, AOH1160 is likely another potential treatment option for combination cancer therapy. [85].

As PCNA is one of the major potential targets for cancer therapy in recent research, efforts lean towards the development of a variety of PCNA inhibiting bioactive materials, including peptides, small molecules, and aptamers [86]. However, chemotherapeutic potency of those developed PCNA inhibitors has been determined mostly via in vitro experiments. Only some of these PCNA inhibitors remain in the effective verification phase in animal models and nothing has been reported for clinical trials to date.

## 3. Targeting PARP1

There are 18 members of the PARP family, and poly (ADP-Ribose) polymerase1gene (PARP1) encodes an enzyme that modifies its substrate proteins by poly(ADP-ribosyl)ation using NAD^+^ [87,88,89,90,91]. PARP1 is about 113 kDa in size, and its domains and structure are well characterized [89,92,93,94,95]. It is comprised of two zinc finger domains that interact with DNA, helix-turn-helix domain, an automodification domain and a catalytic domain. Substrates for PARylation are histone proteins and PARP1 itself [96,97]. PARP1 is involved in DNA single-strand break repair (SSBR) via the base excision repair (BER) pathway, but it is also involved in DSB [87,96,98,99,100,101]. Structural and functional studies have shown that PARP1 accumulates at the site of SSB via its zinc finger domain and interacts with XRCC1, a scaffolding protein that recruits SSBR factors [95,102,103,104]. PARP^-/-^ mice are viable and fertile, indicating that PARP1 is not essential for survival and double knock-out in p53^-/-^ mice results in tumor latency [105]. PARP1 is still considered to be an important factor for genomic maintenance and genotoxic stress response.

PARP1 is overexpressed in multiple cancer types including breast cancer [106,107,108,109], small cell lung cancer [110], nasopharyngeal carcinoma [111], acute myeloid leukemia [112], high-grade epithelial ovarian cancer [113], and colorectal carcinoma [114], often with poor outcomes. The overexpression of the tumor suppressor PARP1 [115] implies non-oncogene addiction of many cancer cells, relying on DDR and repair pathways for survival. There are two mechanisms in which PARP inhibitors can kill HR deficient cancer cells. One is by trapping PARP1 at the site of DNA damage, inhibiting PARylation of substrates and PARP1 remain bound to the lesion. Second mechanism is by increasing SSB. Both scenarios lead to replication fork collapse and/or increased DSB [116,117,118,119]. In 2005, two crucial papers demonstrated the hypersensitivity of *BRCA1/2* deficient tumor cells to PARP1 inhibitors (PARPi) [116,117]. Since then, PARPi is often used in a number of *BRCA1/2* deficient tumors due to synthetic lethality with HR [120,121]. Due to this reason, PARP inhibitors are common cancer therapeutic agents that are heavily investigated. Cells defective in HR rely on NHEJ for DDR, which is an error-prone mechanism and ultimately results in cell death. Main targets for PARPi therapy are *BRCA1/2* mutant or HR defective cancer types, but it is also effective in tumors with RAD51C, RAD51D, and PALB2 mutations [122,123,124,125].

There are several PARPi that are FDA approved including niraparib (MK-4827), Olaparib (AZD-2281), talazoparib (BMN-673) and, rucaparib (AG-014699), that show minimal side-effects [126,127,128]. There are a host of other inhibitors, such as pamiparib (BGB-290), veliparib (ABT-888), CEP-9722, E7016 (GPI-21016), and INO-1001. PARPi causes cancer cell death first by inhibiting its function in SSB, where it leads to the accumulation of DSB. As PARPi is used in cancer with *BRCA1/2* mutations which are defective in HR, upon inhibition, cells must revert to the error-prone NHEJ or cause replication fork stalling [120,129]. Both of these errors require *BRCA1/2* to resolve. Second, it traps the PARP1 enzyme on the chromatin, forming a lesion that requires HR to repair [130]. Talazoparib has one of the lowest IC_50_, with one of the highest efficacies, most likely due to its trapping activity [118].

Although PARPi are effective in many *BRCA1/2* and HR deficient cancers, resistance often develops for a number of reasons. One phenomenon is the restoration of HR, often accomplished by a reversion of *BRCA1/2* mutation and/or epigenetic alteration that re-activates its function [131,132,133,134], or loss of 53BP1 which suppresses NHEJ and allows for ATM-dependent HR [135,136,137,138,139]. Another is by acquiring an alternate mechanism for replication fork protection [140]. Finally, due to the decreased expression levels or enzymatic activity of PARP1 in cancer cells over time, they naturally grow resistant to PARPi [138,141]. Developing resistance to PARPi presents a challenge in HR deficient cancer therapy, and many have sought alternative treatment to overcome this issue. One such option is a combination treatment of PARPi with cytotoxic chemotherapy agents which have been highly effective in treating tumors. Targeting topoisomerase I (TOP1) and PARP1 has shown clinical relevance [142,143]. PARP1 stabilizes the topoisomerase I cleavage complex, providing rationale for inhibiting both targets. Additionally, DNA damaging agents are often used in combination treatments with PARPi [144,145,146,147,148]. Side effects, such as myelosuppression, restricts the full treatment dose of chemotherapeutic agents when combined with PARPi [144]. PARP inhibitor olaparib and CHK1 inhibitor combination treatment will be further discussed in this review.

## 4. Targeting CHK1

CHK1 is part of the serine/threonine protein kinase family and a cell cycle checkpoint protein. It is responsible for G_2_/M checkpoint in response to DNA damage and unreplicated DNA [149]. It is activated by ATR by phosphorylation at serine-345 [149], and inhibits its downstream effector CDC25A by phosphorylation, delaying cell cycle progression in response to DNA double-strand breaks (DSB) [149,150,151]. CHK1 phosphorylates RAD51 at threonine-309 and releases it from *BRCA2*, enhancing its interaction with chromatin [152]. CHK1 also phosphorylates *BRCA2*, which enhances its RAD51 interaction, promoting HR DNA repair [153]. This tumor suppressive function of CHK1 is critical for normal cell survival and is often dysregulated in tumors. Although not heavily discussed in this review, a related checkpoint kinase protein, CHK2, is phosphorylated by ATM, and is also a cell cycle regulator. It inhibits CDC25, thereby preventing entry into mitosis [154]. Although there are several CHK inhibitors that target both CHK1 and CHK2, their IC_50_ differ in many cases, as does their efficacy.

CHK1 is overexpressed in multiple cancer types such as gastric cancer [155], lung adenocarcinoma [156], hepatocellular carcinoma [157], colorectal cancer [158], T-cell acute lymphoblastic leukemia [159], triple-negative breast cancer [160], and nasopharyngeal carcinoma [161]. Since CHK1 is essential for cell survival, cancer cells are often heavily dependent on CHK1, making it an attractive target for cancer therapy. Conversely, elevated levels of CHK1 expression or activating phosphorylation at ser-345 may lead to therapy resistance [162,163]. This is often the case when a cancer cell is addicted to CHK1 and multiple treatment regimens have been used to attempt to overcome this hurdle. On one hand, therapy resistance due to CHK1 enhancement may be problematic, but on the other hand, CHK1 overexpression or addiction in cancer cells with resistance to other drugs provides an alternative treatment option. This synthetic lethality has been taken advantage of in several PARP inhibitor resistant cancer cases, targeting the ATR/CHK1/Wee1 signaling cascade [118]. As we will further explore, targeting CHK1 can either resensitize cells to PARPi, or increase cellular stress, leading to cell death. We have expanded on the usage of PARP inhibitors in the previous section. Often times, cancer cells grow resistant to PARPi, and an alternative treatment becomes necessary. Because PARPi’s accumulate DSB, cancer cells become addicted to the HR pathway or cell cycle checkpoint proteins to override the inhibitory signal and continue to replicate. There are currently many clinical trials that are investigating combination treatments with PARPi and CHK1 inhibitors or the use of CHK1 inhibitors in PARPi or cisplatin resistant cancer patients [118,164,165].

CHK1 as a potential target for cancer therapy was first established in the study of caffeine and its synergistic lethal effect with nitrogen mustard [166,167]. These cytotoxic agents enabled bypass of S-phase arrest into mitosis in p53-deficient cells [168,169]. This led to the development of a kinase inhibitor UCN-01 (7-hydroxystaurosporine), which was identified to function through targeting CHK1 [170,171]. Currently there are several CHK1 inhibitors to date. GDC-0575, LY3300054, MK-8776(SCH-900776), SRA-737 (CCT245737), AZD7762 (no longer in clinical trial), and prexasertib (LY2606368) are some of these drugs. These drugs mainly target replication stress induced by perturbation of CHK1 function [118,172]. Prexasertib in particular, has shown significant potential in regulating the tumor growth of PARPi resistant cases. In the case of high-grade serous ovarian cancer (HGSOC) as well as breast cancer, PARPi are often used as the main line of treatment as well as in maintenance setting after a response to platinum-based chemotherapy [165,173]. Genetic or epigenetic alterations in the HR pathway especially *BRCA1/2* and other Fanconi Anemia related genes are found in approximately 50% of HGSOC cases [165,174,175]. Few PARPi are approved by the FDA for treating cancer patients harboring *BRCA* alterations. Problem arises when patients grow resistant to PARPi, either due to *BRCA1/2* restoration, additional mutation enhancing *BRCA* activity, or other methods of HR restoration. Prexasertib (LY2606368), a CHK1 inhibitor, has shown promising results in combination as well as in mono-therapy along with PARPi in HGSOC cell lines and mouse xenograft model. Parmer et al. demonstrated using a panel of Olaparib resistant HGSOC patient cells, that treatment with prexasertib significantly reduced tumor growth in patient-derived xenograft models [165]. It was effective in both Olaparib sensitive and negative models, as well as in models with or without *BRCA* mutations. Furthermore, synergistic effects of Olaparib and prexasertib combination therapy were observed in both PDX models and established HGSOC cell lines, providing potential alternative treatment options. Other drugs in combination with CHK1 inhibitors such as gemcitabine (chemo)/LY2880070 (NCT02632448) [176]. LY3300054(PD1 inhibitor)/prexasertib (NCT03495323) and olaparib (PARPi)/prexasertib (NCT03057145) that induce replication stress have also been under clinical trial. There are side-effects to CHK1 inhibitors such as in the case of AZD7765, causing such cardio-toxicity that further development was terminated [177]. On the other hand, because CHK1 inhibitors are often used with other chemotoxic agents, lower doses can be effective, which decreases the severity of potential side effects [118,178].

## 5. Targeting Wee1

Wee1 is a serine/threonine G2 checkpoint kinase, and its substrate proteins are CDC1 and CDC2. Its inhibitory phosphorylation on Tyrosine15 of CDC1 prevents cells G2-phase clearance into mitosis when there is DNA damage. Due to its negative regulation of entry into mitosis, it functions as a tumor suppressor in non-malignant cells [179]. Vassilopoulos et al. showed that conditional heterozygous deletion of Wee1 in mice resulted in cell cycle progression while the cells were still under DNA replication, which ultimately caused cancer [180]. This study shows that Wee1 is essential for normal cell cycle progression. Wee1 protects replication forks and chromosome integrity by preventing DNA damage via indirect interaction with MUS81 [181]. Due to this role, Wee1 is considered a marker for replication stress.

Wee1 is found to be overexpressed in hematological tumors such as acute lymphoblastic leukemia (ALL), acute myeloid leukemia (AML), chronic myeloid leukemia (CML), chronic lymphocyte leukemia (CLL), multiple myeloma (MM), and diffuse large B cell lymphoma (DLBCL) [179,182,183,184,185]. It is also overexpressed in solid tumors, such as gastric cancer (GC), malignant melanoma (MM), glioma, ovarian cancer (OC), and colorectal cancer (CC) [179,186,187,188]. Inhibiting Wee1 has been a strategy for cancer targeted therapies, where it has been shown to be effective. For example, ALL cells are dependent on Wee1 for proliferation and survival, and expression level of PKMYT1, a Wee1 family kinase, affects Wee1 inhibitor sensitivity [184]. It functions downstream of the ATR/CHK1 pathway which regulates the DNA damage response and cell cycle during S-phase. Inhibiting Wee1 allows cells to prematurely enter mitosis [189,190]. In this light, Wee1 can be considered as a non-oncogene to which cancer cells are addicted. Inhibiting Wee1 promotes active CDK1-cyclin B1 complex, often resulting in early mitotic entry [191,192]. Targeting Wee1 in combination with DNA damaging agents quickly accumulates DNA damage, resulting in cells death.

AZD1775 (Adavosertib) is a Wee1 inhibitor developed by AstraZeneca. Its IC_50_ is 5.18 nM, inducing DNA damage, G2 checkpoint escape, and early mitotic entry (https://ncats.nih.gov/files/AZD1775). In animal xenograft models, AZD1775 shows enhanced anti-tumor effect with gemcitabine, carboplatin, cisplatin, and other chemotherapy drugs. There are multiple clinical trials testing AZD1775 in combination with these drugs for cancer treatment, including esophageal adenocarcinoma (AJCC), gastroesophageal junction adenocarcinoma (NCT04460937), central nervous system embryonal tumor (NCT02095132), glioblastoma (NCT01849146), cervical carcinoma and endometrioid adenocarcinoma (NCT03345784), advanced malignant solid neoplasm (NCT01827384), pancreatic adenocarcinoma (NCT02194829), ovarian carcinoma (NCT02101775), squamous cell lung cancer (NCT02513563), head and neck squamous cell carcinoma (NCT02585973), colorectal cancer (NCT02906059), advanced acute myeloid leukemia (NCT02666950), and many more.

The combination treatment of AZD1775 and DNA damaging agents has shown promising results in in vitro as well as in vivo, and as shown previously, there are many on-going and completed clinical trials. Recently, Brunner et al. investigated a potential biomarker predicting AZD1775 efficacy in breast cancer. Basal-like breast cancer (BLBC) cell lines were more sensitive to ADZ1775 than luminal types, and low PTEN protein expression level as well as mRNA level correlated with increased sensitivity to the inhibitor [193]. Combination synthetic lethality between Wee1 and the HR pathway was previously reported [194], and cell viability was decreased upon depletion of genes involved in replication stress and HR when treated with AZD1775 [193]. Brunner et al. also showed that NU7441, a DNA-PK inhibitor of NHEJ, and AZD1775 co-treatment synergistically reduced cell viability.

As briefly mentioned above, the ATR/CHK1 pathway is upstream of Wee1 function. Brunner et al. demonstrated that inhibiting ATR and Wee1 simultaneously displays synthetic lethality in BLBC [193]. AZD1775 mono-treatment was compared to combination treatment of ATR inhibitors AZD6738 and AZD1775. In various in vitro and in vivo xenograft studies, only the combination treatment resulted in a prolonged decrease in cell proliferation, DNA replication and cell cycle progression. As many cancer treatment regimens face the issue of resistance, targeting multiple proteins and pathways shows improved results with lower side effects and toxicity [118]. There are currently multiple clinical trials utilizing this strategy inhibiting Wee1 and PARP with Olaparib (NCT04197713, NCT03579316, NCT03330847) in several cancer types.

## 6. Targeting TONSL

**Figure 2:**
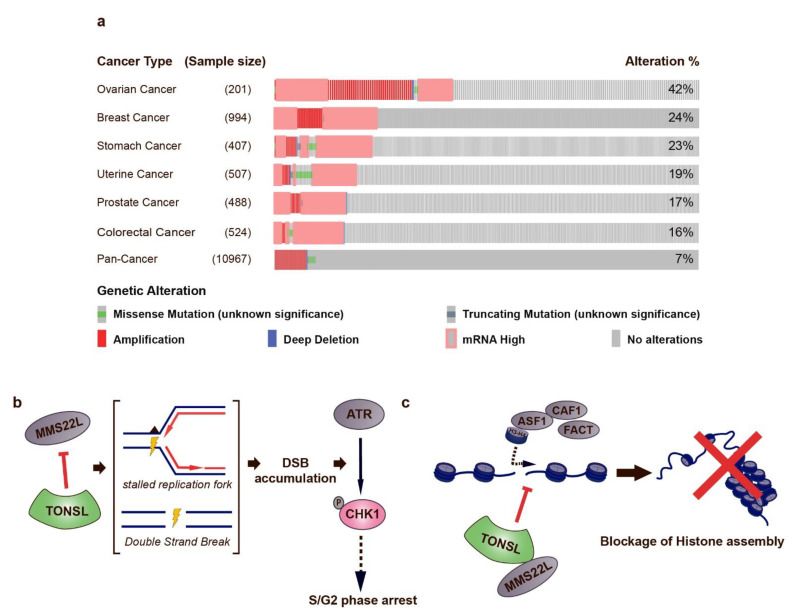
TONSL as a potential novel target protein for targeted cancer therapy. (**a**) TONSL aberration is identified across multiple cancer types. Major aberration was copy number amplification or increase of mRNA expression level. These aberrations were especially high in ovarian and breast cancer. Data was retrieved from The Cancer Genome Atlas and analyzed using cBioPortal.com. (**b**,**c**) Potential mode of action for TONSL inhibitors. b shows the inhibition of DSB repair and replication fork stalling restoration and its involvement in CHK1 phosphorylation. c depicts TONSL’s role in histone assembly. Inhibition of TONSL will result in unstable chromosome, ultimately increasing the genomic instability of cancer cells.

As precision medicine allows for identification of new therapeutic targets, novel targets for cancer therapy are continuously being investigated. This review is focused on non-oncogene addiction, especially genes that are synthetically lethal in the HR pathway, replication stress, and cell cycle check point pathways, here, we discuss a potential new candidate. Tonsuoku-Like DNA Repair Protein (TONSL or NFKBIL2) is a relatively novel gene involved in HR, replication fork repair and chromatin formation [195,196,197,198]. It has been reported that TONSL is overexpressed in hepatocellular carcinoma, and it is implicated in the carcinogenesis of several cancers including lung and esophageal cancer [199,200]. Analysis of TCGA PanCancer studies using cBioPortal.org shows that TONSL is amplified in estimated 7% of all cancer types, 24% in breast cancer, 23% in stomach cancer, and up to 42% in ovarian cancer (Figure 2a, cBioPortal.org).

TONSL is a scaffold protein, interacting with H3/H4 histone protein, ASF1, the MCM complex in the N-terminal domain, and MMS22L in the C-terminal domain [201]. Along with its interaction partners, TONSL is involved in replication stress as well as DNA double-strand break (DSB) repair, especially in the homologous recombination (HR) pathway. Its downregulation results in decreased cell proliferation, increased sensitivity to camptothecin (CPT), replication fork stalling, and increased level of phospho-CHK1 and CHK2 [196,202]. Knockdown of TONSL by siRNA treatment in patient cells from SPONASTRIME dysplasia, a rare weak bone disease caused by hypomorphic mutation of TONSL, reduces RAD51 foci upon CPT treatment, revealing its essential role in RAD51’s ability to load to DNA damage sites by interacting with RPA [202,203].

Experimental data implies targeting TONSL may be effective for cancer therapy. When surveying the structure of TONSL, there is no enzymatic domain and no enzymatic activity has been observed [195,201]. Due to its known activity is through its interaction partners, the most plausible small molecule drug development strategy is likely disrupting the protein-protein interactions (PPI) similar to the PCNA inhibitors described in the previous section. There are several options for utilizing TONSL PPI for small molecule development as it has multiple interaction partners in different pathways. Another reason TONSL may be an attractive target for cancer drug development is due to the different mutation profile and pathway reliance (Figure 2b,c). Although experimentally not yet shown, there is the possibility of synthetic lethality of TONSL inhibition in HR-defective or replication stressed cancer cells, similar to other compounds discussed in this paper. One potential strategy is inhibiting TONSL in *BRCA1/2* deficient cancer, similar to PARPi. A review by Cleary et al. stated that DDR inhibitor target proteins like PARP and Polθ have RAD51 as a pharmacodynamic marker [118]. Previous reports have shown that depletion of TONSL inhibits RAD51 foci, similar to *BRCA2*. Another possible strategy is increasing replication stress by combination treatment of CHK1 inhibitors and TONSL inhibition. Chang et al. has shown that decreased levels of TONSL result in significantly lower BrdU incorporation into DNA, as well as stalled replication forks as shown by DNA fiber assay [202]. In addition, TONSL mutation induced G_2_/M arrest as well as increased phosphorylation of CHK1 [201,202]. If CHK1 is inhibited in combination with TONSL inhibition, it will increase DNA damage and replication stress causing the cell cycle to bypass into mitosis, further burdening the cancer cells. Saredi et al. published a paper solving the structure of TONSL ankyrin repeat domain (ARD) interacting with histone H4 tail, and filed a patent for a small molecule drug development targeting the TONSL-histone protein interaction. [201,204]. The inventors mention that mutation in the ARD domain was identified in multiple cancers types, indicating that this region may be critical for TONSL’s function. In fact, this domain is essential for TONSL-MMS22L accumulation at the site of DNA lesion and stalled replication fork. The inventers tested several peptide compounds blocking the TONSL ARD-histone H4 tail interaction site. For small molecules, about 12.7 million compounds were virtually screened, with the best hit being AG100021 (3-[(3-Aminocyclopentyl) carbonyl]-IH-quinolin-4-one scaffold), about 20% complexed at 20 µM.

Although the TONSL complex is yet to be tested as a target for cancer therapy, the development possibilities are bright. The tools for identifying a hit compound or designing peptide mimetics have already been proven and utilized. Currently, the only patented drug development strategy is utilizing the TONSL-histone PPI site, when in fact TONSL has several other interaction partners. These developments will require the full structure of the protein, but with modern technology, computational modeling can provide clues for other active site options.

## 7. Concluding Remarks

The options for personalized cancer therapy have increased over the past several decades. Since the announcement of the US government investing more resources in precision medicine, the medical community as well as the science community has delved in to developing tools for more precise diagnoses and treatments. Although developing new drugs and biomarkers is essential, overcoming drug resistance is an ever-increasing medical challenge. Developing new drugs with better efficacy is much needed, as well as identifying novel targets to disrupt the tumorigenesis pathway. Utilizing synthetic lethal genes is a very useful strategy to overcome drug resistance in cancer patients, as the cancer cells are still addicted and dependent on several pathways for survival.

In this review article, we have discussed the non-oncogene addiction of cancer cells, especially with well-established target proteins that are synthetically lethal with DDR, DSB repair (including HR and NHEJ pathways), and DNA replication fork stalling. There are several FDA approved small molecule drugs that target PARP1 and CHK1, as well as several in the developmental stage targeting PCNA and Wee1. Conventionally, proteins that are preferred targets for cancer drug development are overexpressed proteins with enzymatic function, but with increased technology, structural simulation has allowed for more diverse small molecule drugs that hinder protein-protein interactions. Such strategies have been used to develop PCNA inhibitors. Here, we suggest the possibility of TONSL as a novel cancer therapeutic target. Its overexpression in multiple cancer types implies that these cancer cells may be dependent on TONSL for survival, and inhibiting its function may be detrimental to tumor growth. Studies of TONSL have shown that downregulating it clearly decreases cell survival and increases drug sensitivity to DNA damaging agents, implying that it may lead to cell death in the context of cancer. To date, no studies have been conducted supporting the non-oncogenic addiction to TONSL in cancer cells, nor its efficacy as a target protein for cancer therapy. However, a patent has been filed for small molecule development targeting the histone interacting domain of TONSL, implying its positive potential as a target protein. Further study will be required to prove its targetability, but the possibility is optimistic when compared to factors that function in the same pathway. It is possible that utilizing TONSL as a novel target may provide alternate options for patients that develop drug resistance to other well-established drugs that target DDR, replication or cell cycle checkpoints, and combinatory treatment studies will be necessary in the future.
